# Socioecologic Factors and Racial Differences in Breast Cancer Multigene Prognostic Scores in US Women

**DOI:** 10.1001/jamanetworkopen.2024.4862

**Published:** 2024-04-03

**Authors:** Ashwini Z. Parab, Angela Kong, Todd A. Lee, Kibum Kim, Edith A. Nutescu, Kristen C. Malecki, Kent F. Hoskins, Gregory S. Calip

**Affiliations:** 1 Department of Pharmacy Systems, Outcomes and Policy, University of Illinois, Chicago; 2 Department of Pharmacy Practice, University of Illinois, Chicago; 3 Center for Pharmacoepidemiology & Pharmacoeconomic Research, University of Illinois, Chicago; 4 School of Public Health, University of Illinois, Chicago; 5 University of Illinois Cancer Center, Chicago; 6 Division of Hematology and Oncology, University of Illinois College of Medicine, Chicago; 7 Titus Family Department of Clinical Pharmacy, University of Southern California, Los Angeles

## Abstract

**Importance:**

Disproportionately aggressive tumor biology among non-Hispanic Black women with early-stage, estrogen receptor (ER)–positive breast cancer contributes to racial disparities in breast cancer mortality. It is unclear whether socioecologic factors underlie racial differences in breast tumor biology.

**Objective:**

To examine individual-level (insurance status) and contextual (area-level socioeconomic position and rural or urban residence) factors as possible mediators of racial and ethnic differences in the prevalence of ER-positive breast tumors with aggressive biology, as indicated by a high-risk gene expression profile.

**Design, Setting, and Participants:**

This retrospective cohort study included women 18 years or older diagnosed with stage I to II, ER-positive breast cancer between January 1, 2007, and December 31, 2015. All data analyses were conducted between December 2022 and April 2023.

**Main Outcomes and Measures:**

The primary outcome was the likelihood of a high-risk recurrence score (RS) (≥26) on the Oncotype DX 21-gene breast tumor prognostic genomic biomarker.

**Results:**

Among 69 139 women (mean [SD] age, 57.7 [10.5] years; 6310 Hispanic [9.1%], 274 non-Hispanic American Indian and Alaskan Native [0.4%], 6017 non-Hispanic Asian and Pacific Islander [8.7%], 5380 non-Hispanic Black [7.8%], and 51 158 non-Hispanic White [74.0%]) included in our analysis, non-Hispanic Black (odds ratio [OR], 1.33; 95% CI, 1.23-1.43) and non-Hispanic American Indian and Alaska Native women (OR, 1.38; 95% CI, 1.01-1.86) had greater likelihood of a high-risk RS compared with non-Hispanic White women. There were no significant differences among other racial and ethnic groups. Compared with non-Hispanic White patients, there were greater odds of a high-risk RS for non-Hispanic Black women residing in urban areas (OR, 1.35; 95% CI, 1.24-1.46), but not among rural residents (OR, 1.05; 95% CI, 0.77-1.41). Mediation analysis demonstrated that lack of insurance, county-level disadvantage, and urban vs rural residence partially explained the greater odds of a high-risk RS among non-Hispanic Black women (proportion mediated, 17%; *P* < .001).

**Conclusions and Relevance:**

The findings of this cohort study suggest that the consequences of structural racism extend beyond inequities in health care to drive disparities in breast cancer outcome. Additional research is needed with more comprehensive social and environmental measures to better understand the influence of social determinants on aggressive ER-positive tumor biology among racial and ethnic minoritized women from disadvantaged and historically marginalized communities.

## Key Points

**Question:**

To what extent are socioeconomic and residential factors associated with higher prevalence of estrogen receptor–positive breast tumors with high-risk recurrence scores (RSs) on the 21-gene assay among minoritized racial and ethnic groups?

**Findings:**

In this cohort study of 69 139 women with breast cancer, non-Hispanic Black and non-Hispanic American Indian and Alaska Native women were more likely to have tumors with high-risk RSs compared with non-Hispanic White women. For non-Hispanic Black women, area-level socioeconomic position, urban residence, and insurance status mediated 17% of the racial difference in the RSs, and racial differences between non-Hispanic Black and non-Hispanic White women in the RSs were observed only among urban women.

**Meaning:**

These findings suggest that disproportionately aggressive breast tumor biology among non-Hispanic Black women may be partially explained by socioecologic factors.

## Teaser

This cohort study examines whether socioeconomic and residential factors mediate racial and ethnic differences in the prevalence of estrogen receptor–positive breast tumors with high-risk gene exression profile.

## Introduction

Poor breast cancer outcomes disproportionately impact racial and ethnic minoritized groups despite concerted efforts to mitigate cancer health disparities.^1,2,3^ Non-Hispanic Black, Hispanic, and non-Hispanic American Indian and Alaskan Native women with early-stage breast cancer have a significantly higher likelihood of disease recurrence and death^4^ compared with non-Hispanic White women. Early-stage tumors that are estrogen receptor (ER) positive and *ERBB2* (formerly *HER2/neu*) (OMIM 164870) negative comprise 70% of breast cancer diagnoses, and a survival disparity according to race and ethnicity among patients with this disease subtype is well documented.^5^

The Oncotype DX 21-gene breast recurrence score (RS) is the most commonly ordered multigene expression biomarker used to estimate the risk of distant metastatic recurrence in women with early-stage, ER-positive/*ERBB2*-negative breast cancer and to identify patients who may derive benefit from adjuvant chemotherapy.^6,7^ Non-Hispanic Black women have a higher prevalence of tumors with a high-risk RS compared with non-Hispanic White women, indicating that biologically aggressive ER-positive tumors disproportionately afflict non-Hispanic Black women.^1,8,9,10^ A recent study found that non-Hispanic Black women in the US are 30% more likely to have a high-risk RS (≥26) on the 21-gene assay compared with non-Hispanic White women.^10^ Notably, the increased prevalence of biologically aggressive tumors among non-Hispanic Black women mediated 20% of the racial disparity in survival after a diagnosis of early-stage, ER-positive breast cancer in the US.^11^

Racial disparity in breast cancer mortality is attributed to socially determined factors, such as greater exposure to social and environmental risk factors and inequitable access to health care. Although studies have found that neighborhood disadvantage, inadequate insurance coverage, and urban residence are associated with worse breast cancer outcomes,^12,13,14,15^ whether these social and environmental factors influence tumor biology among non-Hispanic Black women is not known. Mediation analysis can quantify the relative contributions of factors on the causal pathway that mediate racial difference in the prevalence of high-risk RS and thereby provide insight into the complex and poorly understood interactions between socioecologic context and tumor biology. Previous mediation analyses have focused primarily on the mediating effects of socioeconomic and insurance status on racial disparities in breast cancer survival and prevalence of late-stage diagnoses.^10,16,17,18,19,20,21,22^ Our aim is to provide an in-depth analysis of the complex association between the social and physical environment and breast tumor biology in racial minoritized women. The objective of this study was to examine the mediating influence of area-level socioeconomic position (SEP), insurance status, and urban residence on racial and ethnic differences in the prevalence of a high-risk 21-gene RS in a diverse cohort that is representative of the US population. Race is conceptualized as a social construct for this study.

## Methods

### Study Design and Selection of Participants

This population-based, retrospective, observational cohort study used the Surveillance, Epidemiology, and End Results (SEER) Program Oncotype DX database^23^ to obtain data on the 21-gene RS provided by the Genomic Health Clinical Laboratory through a linkage with invasive breast cancer cases in the SEER registry. We identified women 18 years or older diagnosed with first primary ER-positive/*ERBB2*-negative breast cancer between January 1, 2007, and December 31, 2015, that was stage I to II according to the American Joint Committee on Cancer’s (AJCC’s) *Cancer Staging Manual*, 6th edition, and was negative for axillary lymph node metastases. Information on *ERBB2* status was only available for cases diagnosed in 2010 and later. Women diagnosed from 2010 to 2017 were excluded from the analysis if their tumors were *ERBB2* positive or of borderline status. Our analytical cohort was a complete case analysis. Women whose race or ethnicity was unknown, those who did not receive RS testing, and those who were diagnosed with breast cancer at autopsy or by death certificate only were also excluded. This cohort study was approved by the institutional review board at the University of Illinois at Chicago, which granted a waiver of consent because the study did not involve human participants research, and all data were deidentified. This report follows the Strengthening the Reporting of Observational Studies in Epidemiology (STROBE) reporting guideline.

### Outcome, Exposure, and Covariates

The primary outcome was the RS, which is measured as a continuous variable on a 0- to 100-point scale, with 0 indicating the lowest risk and 100 indicating the highest risk of distant recurrence. Study participants were categorized as having low- or intermediate-risk RSs (range, 0-25) or high-risk RSs (range, 26-100) based on the pivotal validation trial of the RS^24^ and practice guidelines during the study period.^6^ Race and ethnicity were categorized according to SEER recoding of race as non-Hispanic White, non-Hispanic Black, American Indian or Alaska Native, non-Hispanic Asian or Pacific Islander, and Hispanic. The choice of mediator variables and demographic factors adjusted as confounders was informed by the Institute of Medicine’s definition of health care disparities, treating social determinants of health as mediating measures of racial and ethnic inequities. Mediator variables included county-level SEP, insurance status, and rural vs urban residence. The SEP is a composite index derived from principal component analysis of 4 county-level variables extracted from SEER, including median household income (continuous), percentage of households below 150% of the federal poverty line (continuous), percentage of women with less than a high school education (continuous), and percentage of women unemployed (continuous).^11^ The initial component, which captured 74% of the variance across these variables, was selected as the county-level SEP index. The calculated county-level SEP index was categorized into SEER breast cancer population–weighted quintiles (with 1 indicating advantage and 5 indicating disadvantage). Health insurance status was categorized as insured and uninsured or Medicaid. To categorize patients as either urban or rural, we used the 2013 Rural-Urban Continuum Codes. These codes categorize metropolitan counties based on population size and nonmetropolitan counties based on their level of urbanization and proximity to a metropolitan area. Following established rural-urban criteria, we considered patients in counties with codes 1 to 3 as urban, whereas those in counties with codes 4 to 9 were classified as rural.^25^ Demographic and clinical variables were collected from SEER registry data. Individual-level demographic information included age at breast cancer diagnosis, insurance status, and marital status. Clinical information included tumor grade categorized as I, II, III, IV, or unknown; AJCC stage categorized as I or II; progesterone receptor (PR) status characterized as borderline, negative, positive, or unknown; and receipt of radiation therapy and chemotherapy (yes vs no or unknown).

### Statistical Analysis

Descriptive analysis of the study population was performed with the 2-tailed *t* test or Pearson χ^2^ test for continuous and categorical variables, respectively. Nonparametric tests were used for variables with nonnormal distribution. Our approach to conducting mediation analyses followed the product method approach proposed by Baron and Kenny^26^ and later extended for multiple mediators by VanderWeele and Vansteelandt.^27^ There are 4 main assumptions of mediation analyses, including no unmeasured confounding in exposure and outcome association, mediator-outcome association, exposure-mediator association, and no mediator outcome confounder affected by the exposure. This approach avoids potential bias of considering each mediator separately, especially when they are interrelated, and accounts for their collective effect more efficiently. This method estimates the presence of mediation, direct effects, and indirect effects through a series of regression analyses. First, we conducted univariate and multivariable analyses regressing the outcome (high-risk RS) on the exposure (race and ethnicity) and a priori measured confounders that included age and marital status. Second, we conducted univariate and multivariate analyses separately, regressing the mediator of interest (ie, area-level socioeconomic status, insurance status, and rural vs urban residence) on race and ethnicity and a priori measured confounders. Using these estimates, we calculated the natural direct effects (NDE), natural indirect effects (NIE), and total effects (TE) as follows:

*PM* = (*NDE* × [*NIE* − 1]) / (*NDE* × *NIE* − 1).

The direct effect describes the exposure-outcome association that does not include the mediator (θ), and the indirect effect describes the part of the exposure-outcome association that incorporates the mediator (1 − θ).

To assess how much the mediator influences the association between exposure and outcome, we performed a proportion-mediated analysis using the Mediation package in R, version 2022.12.0 + 353 (R Foundation for Statistical Computing). The formula provided represents proportion mediated, which quantifies the proportion of the total effect that can be attributed to the mediating variable. We used the calculated coefficients from the logistic regression model to characterize the outcome variables and the mediator variables. Additionally, we tested for statistical significance of exposure-mediator interaction terms and conducted stratified analyses by our socioecologic factors of interest to evaluate possible effect modification. We calculated the e-value for logistic regression of the exposure-outcome association as part of the sensitivity analysis to measure the minimum strength of association that an unmeasured confounder would need to have with both the exposure and the outcome to fully explain away the observed association. Another sensitivity analysis included only patients with confirmed *ERBB2*-negative tumors who were diagnosed between January 1, 2010, and December 31, 2015. Data analysis was conducted between December 2022 and April 2023. All analyses were conducted using Stata, release 18 and R programming languages (StataCorp LLC). All *P* values were calculated from 2-sided tests, and results were deemed statistically significant at *P* < .05.

## Results

### Characteristics of Study Participants

This study included a total of 69 139 women (mean [SD] age, 57.7 [10.5] years; 6310 Hispanic [9.1%], 274 non-Hispanic American Indian or Alaskan Native [0.4%], 6017 non-Hispanic Asian and Pacific Islander [8.7%], 5380 non-Hispanic Black [7.8%], and 51 158 non-Hispanic White [74.0%]) diagnosed with AJCC stage I to II, axillary node–negative, ER-positive breast cancer (eFigure in Supplement 1). Characteristics of study participants by racial and ethnic category are described in eTable 1 in Supplement 1. More high-grade (grades III- IV) tumors were observed among non-Hispanic Black women (54 [19.4%]) and non-Hispanic American Indian and Alaskan Native women (1245 [19.7%]). Non-Hispanic Black women (1116 [20.7%]), non-Hispanic American Indian and Alaskan Native women (59 [21.5%]), and Hispanic women (1245 [19.7%]) were more likely to live in neighborhoods classified in the lowest SEP quintile compared with non-Hispanic White women (5629 [11.0%]) and non-Hispanic Asian and Pacific Islander (344 [5.7%]). More non-Hispanic Black women (5014 [93.2%]), non-Hispanic Asian and Pacific Islander women (5801 [96.4%]), and Hispanic women (6055 [96.0%]) included in this study tended to live in urban areas compared with non-Hispanic White women (45 377 [88.7%]). Additionally, more non-Hispanic Black women (982 [18.3%]), Hispanic women (1481 [23.5%]), and non-Hispanic American Indian and Alaskan Native women (60 [21.9%]) were enrolled in Medicaid or uninsured compared with non-Hispanic White women (3191 [6.2%]) and non-Hispanic Asian and Pacific Islander women (688 [11.4%]).

### Association of Patient and Tumor Characteristics With High-Risk RSs

More non-Hispanic Black women (939[17.5%]) and non-Hispanic American Indian and Alaskan Native women (49 [17.9%]) had tumors with a high-risk RS compared with non-Hispanic White women (6973 [13.7%]), non-Hispanic Asian and Pacific Islander women (868 [14.5%]), and Hispanic women (874 [13.8%]) (Table 1). Characteristics of study participants according to RS risk category are presented in Table 1. Compared with women with a low- to intermediate-risk RS, the high-risk RS group comprised more high-grade tumors (4574 [47.1%] compared with 6322 [10.7%]), more stage II tumors (3058 [31.5%] compared with 13 305 [22.4%]), and more PR-negative tumors (2574 [26.5%] compared with 3727 [6.3%]). Most women diagnosed with tumors with a high-risk RS received chemotherapy (6353 [65.5%] vs 7038 [11.8%]), and more resided in neighborhoods with low SEP (5393 [55.6%]) compared with participants with low RSs (32 182 [54.1%]).

**Table 1.** Characteristics of Study Participants According to the 21-Gene RS Risk Category

Characteristic	No. (%) of patients		*P* value
	High-risk RS (range, 26-100) (n = 9703)	Low- to intermediate-risk RS (range, 0-25) (n = 59 436)	
Race			
Hispanic	874 (9.0)	5436 (9.1)	<.001
Non-Hispanic American Indian and Alaskan Native	49 (0.5)	225 (0.4)	
Non-Hispanic Asian and Pacific Islander	868 (8.9)	5149 (8.7)	
Non-Hispanic Black	939 (9.7)	4441 (7.5)	
Non-Hispanic White	6973 (71.9)	44 185 (74.3)	
Age group, y			
<45	1195 (12.3)	6395 (10.8)	<.001
45-54	2457 (25.3)	17 135 (28.8)	
55-64	3266 (33.7)	19 168 (32.2)	
65-74	2290 (23.6)	13 883 (23.4)	
75-84	474 (4.9)	2720 (4.6)	
≥85	21 (0.2)	135 (0.2)	
Tumor grade			
I	814 (8.4)	19 192 (32.3)	<.001
II	4186 (43.1)	32 584 (54.8)	
III	4543 (46.8)	6280 (10.6)	
IV	31 (0.3)	42 (0.1)	
Unknown	129 (1.3)	1338 (2.3)	
AJCC stage			
I	6645 (68.5)	46 131 (77.6)	<.001
II	3058 (31.5)	13 305 (22.4)	
PR status			
Borderline	35 (0.4)	62 (0.1)	<.001
Negative	2574 (26.5)	3727 (6.3)	
Positive	7079 (73.0)	55 564 (93.5)	
Unknown	15 (0.2)	83 (0.1)	
Radiotherapy			
No or unknown	4223 (43.5)	22 294 (37.5)	<.001
Yes	5480 (56.5)	37 142 (62.5)	
Chemotherapy			
No or unknown	3350 (34.5)	52 398 (88.2)	<.001
Yes	6353 (65.5)	7038 (11.8)	
Insurance status			
Insured (private or Medicare)	8765 (90.3)	53 972 (90.8)	.14
Medicaid or uninsured	938 (9.7)	5464 (9.2)	
Residence			
Rural	999 (10.3)	5680 (9.6)	.02
Urban	8704 (89.7)	53 756 (90.4)	
Marital status			
Married	6046 (62.3)	37 752 (63.5)	.02
Not married	3316 (34.2)	19 474 (32.8)	
Unknown	341 (3.5)	2210 (3.7)	
Neighborhood socioeconomic position			
1 (Most advantaged)	2171 (22.4)	13 938 (23.5)	.02
2	2139 (22.0)	13 316 (22.4)	
3	1930 (19.9)	11 812 (19.9)	
4	2277 (23.5)	13 163 (22.1)	
5 (Most disadvantaged)	1186 (12.2)	7207 (12.1)	

Abbreviations: AJCC, American Joint Committee on Cancer; PR, progesterone receptor; RS, recurrence score.

### Racial Differences in the RSs by Social and Residential Factors

Compared with non-Hispanic White women, non-Hispanic Black women (odds ratio [OR], 1.33; 95% CI, 1.23-1.43) and non-Hispanic American Indian and Alaskan Native women (OR, 1.38; 95% CI, 1.00-1.86) were more likely to have high RSs after adjusting for age and marital status (Table 2). The association of race with a high-risk RS among non-Hispanic Black women was attenuated and became nonsignificant after adjusting for multiple mediators (area-level SEP, insurance status, and urban residence) and their respective exposure mediator interaction terms (OR, 1.26; 95% CI, 0.92- 1.70). Sensitivity analyses that were restricted to patients with diagnosed in 2010 and later with confirmed *ERBB2-*negative tumors showed results consistent with the findings of the main analyses (eTable 2 in Supplement 1).

**Table 2.** Association of Race and Ethnicity With High-Risk Recurrence Score

Group	Unadjusted model		Multivariable models			
	OR (95% CI)	*P* value	OR (95% CI)^a^	*P* value	OR (95% CI)^b^	*P* value
Hispanic	1.02 (0.94-1.1)	.63	1.02 (0.94-1.1)	.61	1.12 (0.79-1.57)	.51
Non-Hispanic						
American Indian and Alaskan Native	1.38 (1-1.86)	.04	1.38 (1-1.86)	.04	1.64 (0.75-3.29)	.19
Asian and Pacific Islander	1.07 (0.99-1.15)	.09	1.07 (0.99-1.16)	.07	1.28 (0.87-1.83)	.19
Black	1.34 (1.24-1.44)	<.001	1.33 (1.23-1.43)	<.001	1.26 (0.92-1.7)	.13
White	1.0 [Reference]	NA	1.0 [Reference]	NA	1.0 [Reference]	NA

Abbreviations: NA, not applicable; OR, odds ratio; SEP, socioeconomic position.

^a^ For model 1 (adjusted for age and marital status).

^b^ For model 1 plus mediator variables (SEP, insurance, and rurality).

In multivariable analyses of the association between race and ethnicity and individual socioecologic factors (Table 3), we found that non-Hispanic Black, Hispanic, and non-Hispanic American Indian and Alaskan Native women were 2-fold more likely to be uninsured or Medicaid covered and live in low-SEP areas, whereas non-Hispanic Black, non-Hispanic Asian and Pacific Islander, and Hispanic women were more likely to live in urban areas compared with non-Hispanic White women. Mediation analyses revealed that county-level SEP, insurance status, and rural vs urban residence taken together accounted for 7% to 20% of racial and ethnic differences in the prevalence of a high-risk RS (Table 4). However, the contribution of these variables was significant only in non-Hispanic Black women (proportion mediated, 17%; *P* < .001). The magnitude of mediation in non-Hispanic Asian and Pacific Islander and non-Hispanic American Indian and Alaskan Native women who also had numerically higher odds of high-risk RSs was similar despite being not statistically significant. We observed an association with SEP, insurance status, and rural vs urban status; hence, we adjusted for the respective interaction terms with race in the mediation analysis.

**Table 3.** Association of Race and Ethnicity With County-Level Socioeconomic Position, Insurance Status, and Rurality^a^

Group	Residing in most disadvantaged county		Being uninsured or Medicaid enrollee		Living in urban area	
	OR (95% CI)	*P* value	OR (95% CI)	*P* value	OR (95% CI)	*P* value
Hispanic	2.08 (1.94-2.23)	<.001	4.28 (3.99-4.59)	<.001	2.86 (2.52-3.26)	<.001
Non-Hispanic						
American Indian and Alaskan Native	2.28 (1.69-3.02)	<.001	3.56 (2.63-4.76)	<.001	0.42 (0.32-0.57)	<.001
Asian and Pacific Islander	0.52 (0.46-0.58)	<.001	1.95 (1.79-2.13)	<.001	3.24 (2.82-3.73)	<.001
Black	2.17 (2.02-2.34)	<.001	2.49 (2.29-2.69)	<.001	1.67 (1.50-1.87)	<.001
White	1.0 [Reference]	NA	1.0 [Reference]	NA	1.0 [Reference]	NA

Abbreviations: NA, not applicable; OR, odds ratio.

^a^ Multivariable logistic regression models adjusted for age, marital status, and mediator variable indicated.

**Table 4.** Mediation Analysis of Racial and Ethnic Differences in Odds of a High-Risk Recurrence Score by Socioeconomic and Residential Factors^a^

Factor	Finding
Hispanic	
NDE	1.02
NIE	1.00
TE	1.02
Proportion mediated, %^b^^,^^c^	7
Non-Hispanic American Indian and Alaskan Native	
NDE	0.74
NIE	1.07
TE	0.72
Proportion mediated, %^b^^,^^c^	16
Non-Hispanic Asian and Pacific Islander	
NDE	1.08
NIE	0.99
TE	1.08
Proportion mediated, %^b^^,^^d^	20
Non-Hispanic Black	
NDE	0.76
NIE	1.06
TE	0.75
Proportion mediated, %^b^^,^^e^	17

Abbreviations: NDE, natural direct effect; NIE, natural indirect effect; TE, total effect.

^a^ Compared with non-Hispanic White women.

^b^ Proportion mediated = [NDE × (NIE − 1)/(NDE × NIE − 1)].

^c^ *P* = .80.

^d^ *P* = .60.

^e^ *P* < .001.

Sensitivity analyses with e-values suggested that a potential unmeasured confounder would need to be associated with both the exposure and outcome with a risk ratio of 2 (or an OR of 2) to fully explain the observed association between race and high-risk RSs in non-Hispanic Black women in the absence of a true association (eTable 3 in Supplement 1). Sensitivity analysis restricted to patients diagnosed in 2010 to 2015 showed a similar crude association between race and high-risk RS, but no significant attenuation in the OR was seen after mediator adjustment in non-Hispanic Black women.

### Urban Residence and Racial Difference in the RS

To better understand rural vs urban residence as a mediator of the association between non-Hispanic Black race and a high-risk RS, we performed analyses stratified by rurality status for non-Hispanic Black and non-Hispanic White women (Table 5). Stratified models adjusted for age, marital status, SEP, and insurance status showed that non-Hispanic Black race was associated with high-risk RSs among women living in urban areas (OR, 1.35; 95% CI, 1.24-1.46), but no statistically significant difference was observed among women living in rural areas (OR, 1.05; 95% CI, 0.77-1.41). The association of non-Hispanic Black race with a high-risk RS was fully attenuated in an unstratified model adjusted only with an interaction term for race × rurality (OR, 1.09; 95% CI, 0.81-1.44). Taken together, these results suggest that the association of non-Hispanic Black race and a high-risk RS differs for urban and rural non-Hispanic Black women, although the interaction term (race × rurality) was not statistically significant (*P* = .14 for interaction; OR, 1.27; 95% CI, 0.94-1.71).

**Table 5.** Association of Black Race and High-Risk Recurrence Score According to Socioeconomic Factors

Stratification	Covariates in multivariable model	OR (95% CI)^a^	*P* value
Urban residence	Age, marital status, SEP, insurance	1.35 (1.24-1.46)	<.001
Rural residence	Age, marital status, SEP, insurance	1.05 (0.77-1.41)	.74
Insured	Age, marital status, SEP, rural	1.38 (1.27-1.50)	<.001
Medicaid or uninsured	Age, marital status, SEP, rural	1.06 (0.86-1.29)	.60
SEP 1 (advantaged)	Age, marital status, rural, insurance	1.67 (1.33-2.08)	<.001
SEP 5 (disadvantaged)	Age, marital status, rural, insurance	1.10 (0.91-1.33)	.31

Abbreviations: OR, odds ratio; SEP, socioeconomic position.

^a^ The reference category is non-Hispanic White race.

## Discussion

To our knowledge, this is the first study to quantify the association between individual-level and contextual disadvantage and urban residence as mediators of racial differences in the prevalence of a high-risk gene expression profile in early-stage, ER-positive breast cancer. The results demonstrated that racial or ethnic differences in the RSs between non-Hispanic White and racial and ethnic minoritized women are not limited to non-Hispanic Black women; both non-Hispanic Black and non-Hispanic American Indian and Alaskan Native patients with breast cancer were 30% more likely to have a high-risk RS compared with non-Hispanic White individuals. Racial differences in the RS were partially mitigated after adjusting for health insurance status, neighborhood disadvantage, and urban residence among non-Hispanic Black women. The analysis found a modest degree of mediation (17%) of the racial difference in the prevalence of a high-risk RS by these socioeconomic and residential factors among non-Hispanic Black women. This finding indicates that socioeconomic deprivation and urban life exposures that are more pervasive in Black communities contribute to disproportionately aggressive tumor biology among non-Hispanic Black women. Notably, in the analysis stratified by rurality, non-Hispanic Black women living in urban areas had higher odds of developing tumors with high-risk RS, whereas that association was not seen in patients living in the rural areas. The study findings suggest that socioecologic context is the main factor underlying biologically aggressive ER-positive tumors in non-Hispanic Black women, although we were unable to examine whether ancestry-related risk alleles act as an effect modifier. Our findings have important implications for future research on the biological mechanisms underlying breast cancer disparities and support a social-genomic approach^28^ to investigating this public health priority.

Socioeconomic position considers both social and economic factors as well as social status and is a well-established factor associated with health inequalities.^29,30,31^ Residing in disinvested, underresourced areas can adversely affect health due to substandard housing, unfavorable social environments, and limited health care access. As results from this analysis suggest, persistent racial discrimination in the US has contributed to a higher proportion of Black, Hispanic, and American Indian populations living in communities with high disadvantage. Lower-economic and highly disadvantaged communities also have a greater number of social and environmental stressors, including higher environmental burden from inadequate housing, higher traffic density, increased industrial pollution, higher crime rates, and poorer access to resources such as healthy food, green space, and medical care.^31,32^ Existing research underscores the notion that environmental and social factors can imprint modifications on the epigenome and subsequently influence gene expression, thus contributing to observed disparities.^33,34^ To date, few studies have examined the potential role that social and environmental stressors from contextual disadvantage may have in contributing to more aggressive and deadly tumor biology.

Given the complexity and intricate nature of gene-environment interactions, it is critical to acknowledge even a modest level of mediation by a few socioecologic factors on disproportionately aggressive tumor biology among Black women.^35,36,37,38^ It is plausible that using more comprehensive and nuanced measures of social determinants of health would explain a greater proportion of this racial difference. The SEP index used in this study was derived from county-level data. Perhaps more granular data at the census tract or block level would provide more insightful information. The SEER data set does not include data that would allow us to measure the effects of genetic ancestry, which prevents us from drawing any inferences about ancestry-related risk alleles that may act as effect modifiers. Previous literature^39,40^ has hypothesized that one of the ways neighborhood socioeconomic disadvantages adversely affect tumor biology is through a correlation with increased obesity. A recent study by Gordon et al^41^ offered evidence of this association by finding that a unit increase in insulin resistance increased the odds of a high-risk RS by 30%. Our study was constrained by the limited availability of measures for obesity and stress, which are presumed to be important factors through which the mediators influence the outcome.

### Strengths and Limitations

There are several strengths of this study, including use of population-based data spanning multiple US states and geographic regions, which increases representation of diverse racial and ethnic groups and allows detailed analysis of residential and socioeconomic variables reflecting the US population. Moreover, we used a composite measure encompassing multiple domains comprising SEP. We also included an individual measure of SEP (insurance status), which increases our ability to measure disadvantage compared with area-level variables alone.^11^ The linkage of SEER data with data from Genomic Health provides a genomic marker of tumor biology that is more robust than traditional histopathologic features.^11^ This study uses mediation analysis to explore how race understood as a social construct can impact tumor biology. The multimediator analysis attempts to explain the disproportionate prevalence of high-risk RSs among Black women as the embodiment of social disadvantage through the accrual of a constellation of socioecologic factors.

This study also has some limitations. The study was restricted to individuals who underwent RS testing, potentially introducing selection bias. We calculated the weighted socioeconomic quintile for all patients with breast cancer, regardless of whether they received the test. We observed that patients with higher socioeconomic status were more likely to be included in the study sample, consistent with previous studies^42,43^ finding testing disparities. It excludes tumors tested with genomic biomarkers other than the 21-gene RS. Additionally, this study only evaluates the exposure to social environments based on the patient’s residence at the cancer diagnosis, and we could not account for environmental exposures before breast cancer diagnosis. Hence, patients’ exposure at the time of cancer diagnosis captured in this study may not necessarily reflect their long-term exposures. Due to smaller sample sizes in non-Hispanic American Indian and Alaskan Native women and a less substantial amount of disparity in high-risk RSs among non-Hispanic Asian and Pacific Islander women, we were not able to evaluate the role of socioecologic factors in these disparities. Further research to evaluate these possible disparities is warranted. Furthermore, the SEER registry did not contain information on *ERBB2* status before 2010. The SEER data set lacks information on insurance status before 2007; we had to exclude those cases from the analysis because insurance status was one of the mediators being studied. Finally, we cannot exclude the possibility of selection bias in the study cohort because RS data are only available for patients whose treating oncologist ordered the test.

## Conclusions

This study demonstrates for the first time, to our knowledge, the influence of socioecologic context on a genomic biomarker that reflects the biology of ER-positive breast tumors. The study highlights the fact that the downstream effects of structural racism are not limited to inequities in health care; they also drive biologically aggressive tumor phenotypes. These effects are most clearly seen among non-Hispanic Black women, although the problem extends to other racial and ethnic minoritized groups (eg, non-Hispanic American Indian and Alaskan Native women). The finding that among non-Hispanic Black women only those with urban residence have a disproportionate prevalence of biologically aggressive tumors confirms the central role of contextual factors as the root cause of this disparity. However, future research should examine the possibility that ancestry-related genetic factors may act as effect modifiers. Additional research is needed with more comprehensive and nuanced measures of the social and environmental exposures that urban non-Hispanic Black and non-Hispanic American Indian and Alaskan Native women are subjected to. Mechanistic studies are also needed to identify potential targets for interventions that improve outcomes for racial and ethnic minoritized women with ER-positive breast cancer.

## Notes

Supplement 1. **eFigure.** CONSORT Diagram **eTable 1.** Characteristics of Study Participants **eTable 2.** Sensitivity Analysis of Association of Race/Ethnicity With Odds of High-Risk Recurrence Score for Patients Diagnosed in 2010-2015 **eTable 3.** Mediational E Value

Supplement 2.
Data Sharing Statement
