# Supplementary material for: Socioecologic Factors and Racial Differences in Breast Cancer Multigene Prognostic Scores in US Women
Source: JAMA Netw Open. 2024 Apr 3;7(4):e244862. doi: 10.1001/jamanetworkopen.2024.4862 (PMC10993076; doi:10.1001/jamanetworkopen.2024.4862)
Supplement: Supplement 1. — eFigure. CONSORT Diagram eTable 1. Characteristics of Study Participants eTable 2. Sensitivity Analysis of Association of Race/Ethnicity With Odds of High-Risk Recurrence Score for Patients Diagnosed in 2010-2015 eTable 3. Mediational E Value [file jamanetwopen-e244862-s001.pdf]

Supplementary Online Content

Parab AZ, Kong A, Lee TA, et al. Socioecologic factors and racial differences in breast cancer multigene prognostic scores of US women. *JAMA Netw Open*. 2024;7(4):e244862. doi:10.1001/jamanetworkopen.2024.4862

eFigure. CONSORT Diagram

eTable 1. Characteristics of Study Participants

eTable 2. Sensitivity Analysis of Association of Race/Ethnicity With Odds of High-Risk Recurrence Score for Patients Diagnosed in 2010-2015

eTable 3. Mediational E Value

This supplementary material has been provided by the authors to give readers additional information about their work.

© 2024 Parab AZ et al. *JAMA Network Open.*

eFigure 1. CONSORT diagram of study cohort selection

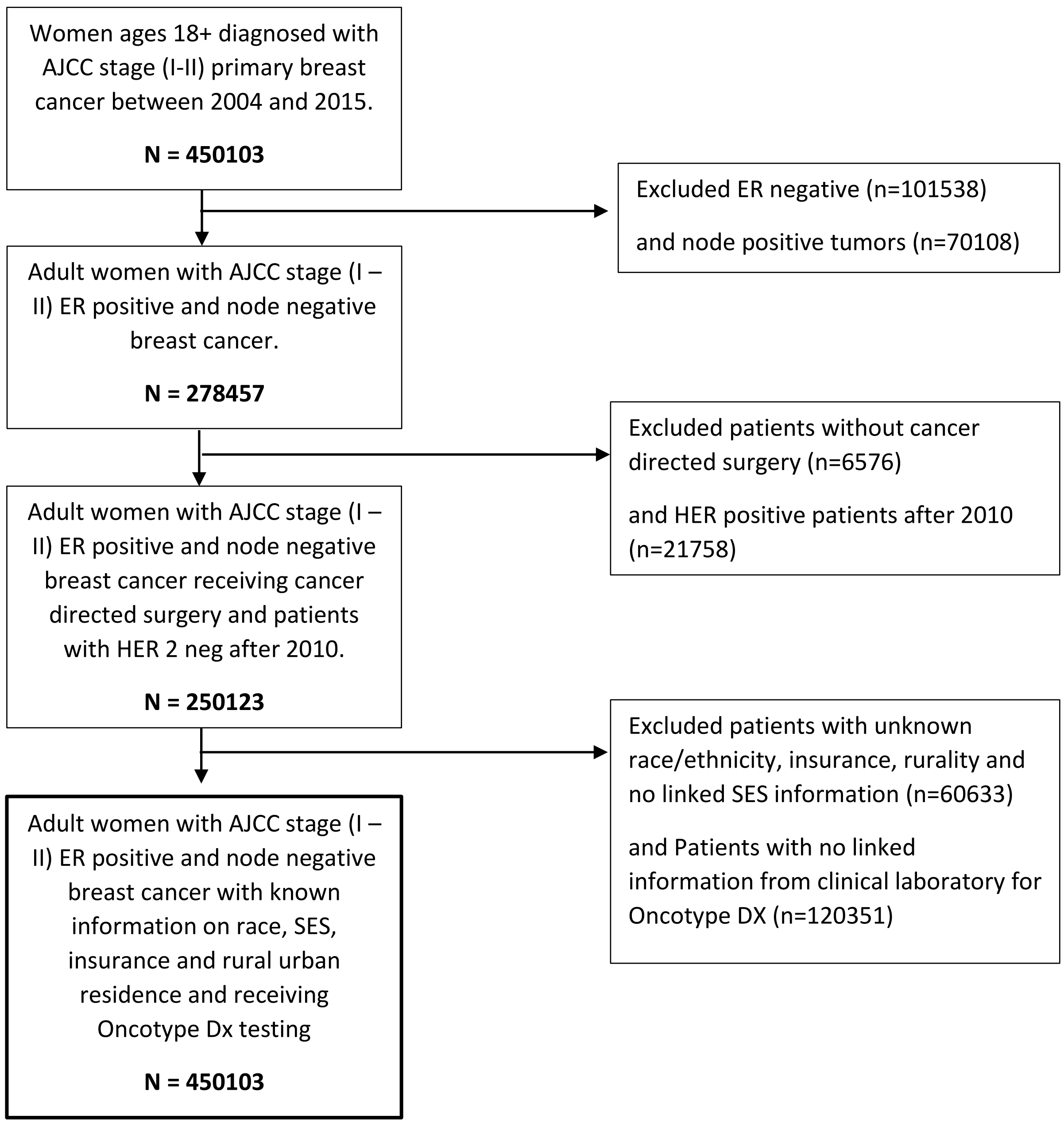

© 2024 Parab AZ et al. *JAMA Network Open.*

eTable 1. Characteristics of study participants

	Non-Hispanic White		Non-Hispanic Black		Non-Hispanic American Indian or Alaskan Native		Non-Hispanic Asian or Pacific Islander		Hispanic		P value
	(n= 51158)		(n= 5380)		(n= 274)		(n= 6017)		(n= 6310)		
	n	%	n	%	n	%	n	%	n	%	
**Age**	58.33 [10.29]		57.13 [10.82]		56.74[9.69]		54.84[10.54]		55.48[10.66]		<0.01
<45 years	4820	(9.4)	687	(12.8)	29	(10.6)	1065	(17.7)	989	(15.7)	
45-54 years	13983	(27.3)	1522	(28.3)	80	(29.2)	1955	(32.5)	2052	(32.5)	
55-64 years	16977	(33.2)	1716	(31.9)	102	(37.2)	1781	(29.6)	1858	(29.4)	
65-74 years	12706	(24.8)	1186	(22.0)	57	(20.8)	1048	(17.4)	1176	(18.6)	
75-84 years	2552	(5.0)	254	(4.7)	6	(2.2)	162	(2.7)	220	(3.5)	
>=85 years	120	(0.2)	15	(0.3)	0	0.0	6	(0.1)	15	(0.2)	
**Grade**											<0.01
Grade I	15282	(29.9)	1365	(25.4)	67	(24.5)	1627	(27.0)	1665	(26.4)	
Grade II	27087	(52.9)	2847	(52.9)	149	(54.4)	3243	(53.9)	3444	(54.6)	
Grade III	7619	(14.9)	1041	(19.3)	52	(19.0)	1046	(17.4)	1065	(16.9)	
Grade IV	46	(0.1)	5	(0.1)	2	(0.7)	8	(0.1)	12	(0.2)	
Unknown	1124	(2.2)	122	(2.3)	4	(1.5)	93	(1.5)	124	(2.0)	
**AJCC stage**											<0.01
I	39597	(77.4)	3964	(73.7)	219	(79.9)	4324	(71.9)	4672	(74.0)	
II	11561	(22.6)	1416	(26.3)	55	(20.1)	1693	(28.1)	1638	(26.0)	
**PR Status**											<0.01
Borderline	77	(0.2)	5	(0.1)	1	(0.4)	4	(0.1)	10	(0.2)	
Negative	4589	(9.0)	613	(11.4)	22	(8.0)	533	(8.9)	544	(8.6)	
Positive	46427	(90.8)	4752	(88.3)	249	(90.9)	5470	(90.9)	5745	(91.0)	
Unknown	65	(0.1)	10	(0.2)	2	(0.7)	10	(0.2)	11	(0.2)	
**ERBB2 Status**											<0.01
Negative	37552	(73.4)	4087	(76.0)	219	(79.9)	4641	(77.1)	4925	(78.1)	
Unknown (diagnosed prior to 2010)	13606	(26.6)	1293	(24.0)	55	(20.1)	1376	(22.9)	1385	(21.9)	
**Oncotype RS**											<0.01
High risk	6973	(13.6)	939	(17.5)	49	(17.9)	868	(14.4)	874	(13.9)	
Low/Intermediate risk	44185	(86.4)	4441	(82.5)	225	(82.1)	5149	(85.6)	5436	(86.1)	
**Chemotherapy**											<0.01

© 2024 Parab AZ et al. *JAMA Network Open.*

No/Unknown	41542	(81.2)	4188	(77.8)	213	(77.7)	4776	(79.4)	5029	(79.7)	
Yes	9616	(18.8)	1192	(22.2)	61	(22.3)	1241	(20.6)	1281	(20.3)	
**Insurance status**											<0.01
Insured (Private or Medicare)	47967	(93.8)	4398	(81.7)	214	(78.1)	5329	(88.6)	4829	(76.5)	
Medicaid or Uninsured	3191	(6.2)	982	(18.3)	60	(21.9)	688	(11.4)	1481	(23.5)	
**Residence**											<0.01
Rural	5781	(11.3)	366	(6.8)	61	(22.3)	216	(3.6)	255	(4.0)	
Urban	45377	(88.7)	5014	(93.2)	213	(77.7)	5801	(96.4)	6055	(96.0)	
**Marital status**											<0.01
Married	33618	(65.7)	2103	(39.1)	133	(48.5)	4220	(70.1)	3724	(59.0)	
Not Married	15661	(30.6)	3059	(56.9)	123	(44.9)	1618	(26.9)	2329	(36.9)	
Unknown	1879	(3.7)	218	(4.1)	18	(6.6)	179	(3.0)	257	(4.1)	
**Neighborhood Socioeconomic position**											<0.01
1 (most advantaged)	13145	(25.7)	502	(9.3)	60	(21.9)	1753	(29.1)	649	(10.3)	
2	12670	(24.8)	1272	(23.6)	61	(22.3)	575	(9.6)	877	(13.9)	
3	9948	(19.4)	835	(15.5)	44	(16.1)	1635	(27.2)	1280	(20.3)	
4	9766	(19.1)	1655	(30.8)	50	(18.2)	1710	(28.4)	2259	(35.8)	
5 (most disadvantaged)	5629	(11.0)	1116	(20.7)	59	(21.5)	344	(5.7)	1245	(19.7)	

Abbreviations: NHW, non-Hispanic White; NHAIAN, non-Hispanic Alaskan Native/American Native; NHAPI, non-Hispanic Asian/Pacific Islander; NHB, non-Hispanic Black; RS, Recurrence Score; PR, progesterone receptor

© 2024 Parab AZ et al. *JAMA Network Open.*

eTable 2: Sensitivity Analysis of Association of Race/Ethnicity with Odds of High Risk Recurrence Score for Patients Diagnosed 2010-2015[1]

	Crude model		Adjusted models			
			Model 1		Model 1 + Mediator variables	
	OR (95% CI	p-value	OR (95% CI	p-value	OR (95% CI	p-value
NHW	[Reference]	NA	[Reference]	NA	[Reference]	NA
Hispanic	1.02 [0.93,1.11]	0.73	1.01 [0.93,1.11]	0.77	0.99 [0.63,1.5]	0.96
NHAIAN	1.36 [0.94,1.92]	0.09	1.36 [0.94,1.91]	0.09	1.21 [0.47,2.75]	0.67
NHAPI	1.07 [0.98,1.17]	0.12	1.07 [0.98,1.17]	0.13	1.21 [0.77,1.84]	0.38
NHB	1.32 [1.21,1.44]	<0.001	1.31 [1.2,1.43]	<0.001	1.4 [0.97,1.97]	0.06

eTable 2. Association of race/ethnicity and high-risk recurrence score among patients diagnosed from 2010-2015 with tumors that are confirmed as ERBB2-negative in multivariable models adjusted for age and marital status only (model 1), or with further adjustment for mediator variables of socioeconomic position, insurance status and rural/urban residence

[1]All participants have ERBB2-negative tumors.

Abbreviations: OR, odds ratio; CI, confidence interval; NHW, non-Hispanic White; NHAIAN, non-Hispanic Alaskan Native/American Native; NHAPI, non-Hispanic Asian/Pacific Islander; NHB, non-Hispanic Black

© 2024 Parab AZ et al. *JAMA Network Open.*

eTable 3: Evaluation of E value to Assess the Impact of Unmeasured Confounder in Exposure Outcome Association.

	Crude model<br>OR (95% CI)	Mediational E value
NHW	1[Reference]	
Hispanic	1.02 [0.94,1.1]	1.16
NHAIAN	1.38 [1,1.86]	2.10
NHAPI	1.07 [0.99,1.15]	1.34
NHB	1.34 [1.24,1.44]	2.01

E value is a measure that assesses the strength of the potential unmeasured confounders needed to explain away the observed exposure and outcome association. Abbreviations: OR, odds ratio; CI, confidence interval; NHW, non-Hispanic White; NHAIAN, non-Hispanic Alaskan Native/American Native; NHAPI, non-Hispanic Asian/Pacific Islander; NHB, non-Hispanic Black

© 2024 Parab AZ et al. *JAMA Network Open.*
