# Supplementary material for: Socioecologic Factors and Racial Differences in Breast Cancer Multigene Prognostic Scores in US Women
Source: JAMA Netw Open. 2024 Apr 3;7(4):e244862. doi: 10.1001/jamanetworkopen.2024.4862 (PMC10993076; doi:10.1001/jamanetworkopen.2024.4862)
Supplement: Supplement 2. — Data Sharing Statement [file jamanetwopen-e244862-s002.pdf]

Data Sharing Statement

Parab. Socioecologic Factors and Racial Differences in Breast Cancer Multigene Prognostic Scores in US Women. *JAMA Netw Open*. Published April 03, 2024.
doi:10.1001/jamanetworkopen.2024.4862

Data
Data available: No

Additional Information
Explanation for why data not available: The data used for our study is from the Surveillance, Epidemiology and End Results Program of the NIH/NCI, available publicly through a limited data use agreement.
